# Environmental Factors Affecting Growth and Occurrence of Testicular Cancer in Childhood: An Overview of the Current Epidemiological Evidence

**DOI:** 10.3390/children4010001

**Published:** 2017-01-05

**Authors:** Fabrizio Giannandrea, Stefania Fargnoli

**Affiliations:** 1 Occupational Health Unit, Local Health Authority, ASL 2 Abruzzo Lanciano-Vasto-Chieti; S.S. Annunziata University Hospital, 66100 Chieti, Italy; 2 Department of Public Health and Infectious Diseases, University of Rome Sapienza, 00185 Rome, Italy; stefaniafargnoli@hotmail.it

**Keywords:** environmental factors, testicular cancer, childhood, child growth, child development

## Abstract

Testicular cancer (TC) is the most frequently occurring malignancy among adolescents and young men aged 15–34 years. Although incidence of TC has been growing over the past 40 years in several western countries, the explanations for this increase still remain uncertain. It has been postulated that early life exposure to numerous occupational and environmental estrogenic chemicals, such as endocrine-disrupting chemicals (EDCs), may play a contributing role in the etiology of TC, but the subject is still open to additional investigation. Recently, it has also been suggested that prenatal and postnatal environmental exposures associated with child growth and development might also be involved in TC progression. This review of current epidemiological studies (2000–2015) aims to identify environmental factors associated with TC, with a particular focus on infancy and childhood factors that could constitute a risk for disease development. It may also contribute towards recognizing gaps in knowledge and recent research requirements for TC, and to point out possible interactions between child growth and development in relation to prenatal and postnatal environmental exposures.

## 1. Introduction

Testicular cancer (TC) is the most frequently occurring malignancy in adolescents and young men aged 15–34 years. Its incidence has been growing over the past 40 years in several western countries [1,2,3], although the reasons for this increase are not completely elucidated. The most consistently identified risk factor associated with TC is cryptorchidism, which increases a man’s risk of TC development by nearly five-fold [1,2,3]. Familial TC is also an established risk factor for developing the disease. Studies have estimated that brothers of TC patients have an eight- to 10-fold increased risk of acquiring TC, whereas the fathers/sons have a four- to six-fold increase in risk [1,2,3].

The incidence peak of TC among young adults suggests that contributing factors could play a role at an early stage of life [1,2,3]. Although it has been suggested for decades that increases in endogenous estrogen concentrations in pregnancy and/or prenatal exposures to several occupational and environmental estrogenic substances such as endocrine-disrupting chemicals (EDCs), are mainly responsible for its etiology [4,5], this theory is still debated. Recent research has suggested that environmental exposures occurring in infancy and childhood may also contribute to the progression of TC, and that factors related to child growth might be deeply involved in TC development [6,7].

This review aims to identify risk factors associated with TC, with a particular attention to infancy and childhood risk factors, to highlight gaps in knowledge and research for TC and to point out possible interactions between child growth and development, in relation to prenatal and postnatal environmental exposures. The studies included in this review were identified in the PubMed database, searched with the keywords: “environmental factors”, “testicular cancer”, “childhood”, “child growth”, and “child development”. This review is based primarily on studies conducted within the past 15 years (2000–2015), although research from older studies were also mentioned within the newer studies.

## 2. Testicular Germ Cell Development in the Infancy Period

**Figure 1:**
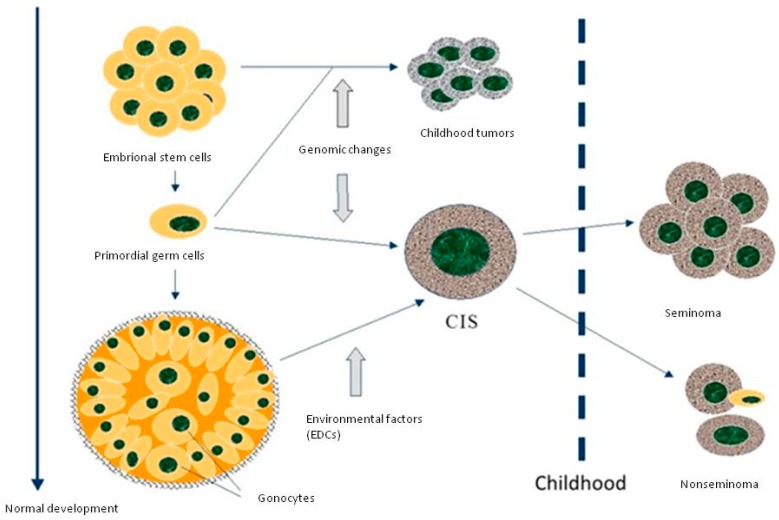
Histogenesis of testicular germ cell tumors.

According to several histopathological studies, TC originated from carcinoma in situ (CIS) cells derived from primordial germ cells [8]. CIS cells are morphologically similar to gonocytes, which express various immunohistochemical markers in common, such as placental-like alkaline phosphatase [9] (a classical marker of primordial germ cells), and KIT (a stem cell factor receptor). The mechanism underlying the progression of CIS towards a malignant form has not yet been explained in detail. The progression of gonocytes into infantile spermatogonia is an ongoing process, which starts approximately at five months of pregnancy and continues in the infancy period until around six to nine months of age [9]. The beginning of the neoplastic conversion is most likely caused by an alteration in the infancy microenvironment of primordial germ cells due to hormones and paracrine factors (Figure 1). Any disturbance in hormonal stability may interfere with the transformation of primordial germ cells or gonocytes into infantile spermatogonia. It has therefore been postulated that a high exposure to estrogens or EDCs might be responsible for the rise of these cells [9]. This microenvironmental instability may also be produced by a genetic defect, in particular an androgen insensitivity [10]. Additionally, an increase in TC incidence rates is seen after puberty, most likely activated by the significant change of sex hormone production associated with child growth [11].

## 3. Time Incidence Trends of Testicular Cancer (TC): Age-Specific Differences

Over the past 40 years, a steady increase in the incidence rates of TC in men has been reported in most western populations, such that in some countries, TC is now the most frequent malignancy among young men aged 15–34 years old. Explanations for this rise have not yet been well elucidated, although improved diagnostic procedures may be partially responsible. In Denmark, the rate of increase of testicular cancer was about 2.6% per year [3]. In Norway, the age-standardized incidence rates for TC more than tripled over just 40 years, with an increase from 2.7 per 100,000 in 1955 to 8.5 per 100,000 in 1992 [2,3].

During a similar time-period in the United States, the overall incidence rates of TC in men rose more than 44%, from 3.4 cases per 100,000 in 1973–1978, to 4.8 per 100,000 between 1994 and 1998. While incidence rates have increased, the type of cancer has remained steady, as no differences in increase have been observed between seminoma and non-seminoma [12]. Although childhood testicular cancer has been less studied, the incidence rates over time have remained constant in Denmark, Norway, Sweden and in the United States [13], which could indicate that the incidence of TC in adulthood is influenced by factors (either prenatal or childhood exposures), that are different than those determining the trends in childhood.

**Figure 2:**
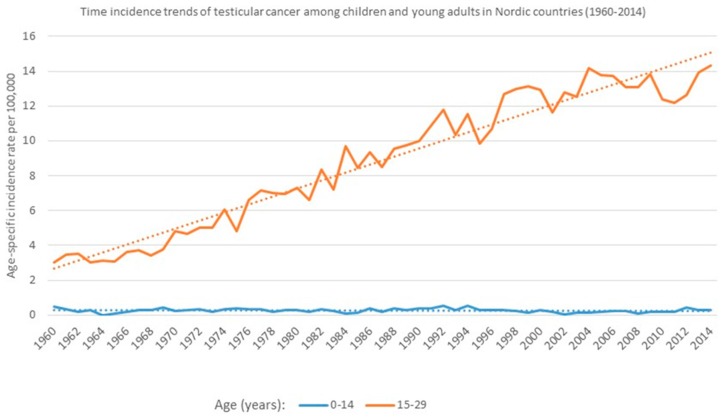
Age-specific time incidence rates of testicular cancer (TC) among children and young adults (age-adjusted according to the world population) in the period 1960–2014, of all Nordic countries (Sweden, Denmark, Finland, Norway and Iceland). Analyses based on data obtained from NORDCAN, 2016.

According to NORDCAN (International Agency for Research on Cancer (IARC)) available at http://www-dep.iarc.fr/NORDCAN/english/frame.asp), the 1960–2014 age-specific incidence rate trends for testicular cancer in Nordic countries (Sweden, Denmark, Finland, Norway and Iceland) in the 0–14 age group of children, have remained consistent and low. This is in direct contrast to the steadily increasing trends observed among young adults over this period (15–29 years old; Figure 2). It is therefore reasonable to postulate that age-specific environmental exposures might play a role in testicular cancer rates. Additionally, data of men 30 years or older also indicate similar increasing trends in the incidence of testicular cancer (data not shown). Therefore, although research on causes of TC in the last few decades have focused mainly on in utero exposures, it is reasonable to speculate that additional infant and child environmental exposures could also be responsible for the incidence rate difference between adults and children.

## 4. Domestic and Residential Exposures to Pesticides in Childhood

Only a few studies have evaluated the relationship between self-reported domestic and residential use of pesticides and TC rates. Studies on serum concentrations of pesticides, mainly dichloro-diphenyl-trichloroethane (DDT) and its metabolite, dichloro-diphenyl-trichloroethylene (p,p’-DDE), are discussed further in Section 7.1. Although one study based on self-reported exposure has showed an increased risk of TC and indoor use of insecticide [14].

Rural area residency in childhood is often used as an indirect indicator of possible environmental exposure to pesticides [15,16,17,18,19]. However, except for one Italian study that showed an association with rural residency and TC [14], only inadequate associations with TC risk have been reported among persons living in a rural area during childhood and adolescence [15,16,17,18,19]. Additionally, among studies focusing on occupational parental exposure, an increased risk has been observed with childhood residence in a Danish high-nitrate area [15], while a Swedish study on children of pesticide applicators was inconclusive. Due to the currently limited and inconclusive data at hand, further studies on domestic and residential exposure should be undertaken in future investigations.

## 5. Diet and Nutrition in Childhood

The idea that the diet of children might be associated with TC originated from several epidemiological investigations of increasing trends in the incidence of TC since the beginning of the 20th century, with the only exception for birth cohorts of men born during World War II, which corresponds to a period when food accessibility had been restricted [20]. It is well-known, from both animal and human studies, that food restriction during childhood may decrease cancer risk later in life [21,22].

TC has also been associated with childhood consumption of dairy products [23,24,25,26], most likely due to the fact that many dairy products (mainly cheese and milk), contain several sex hormones that had been given to the animal, such as progesterone and estrogen [25]. In an ecological study comparing TC rates in 42 countries and their dietary practices, Ganmaa et al. found that self-reported consumption of cheese during pregnancy or childhood, in the years 1961–1965, was highly correlated with the incidence of TC later in adulthood and that combined consumption of milk and cheese during childhood were significantly associated with incidence rates of TC [25]. Garner et al. also observed that elevated intake of dairy products two years before the diagnosis of TC was strongly associated with the risk of the disease [27]. Additionally, Davies et al. reported in their case-control study, a higher intake of milk during childhood and adolescence among TC cases compared with healthy controls [24]. Another study, by Stang et al., childhood and adolescent intake of dairy products, in particular galactose and milk consumption, was found to be a risk factor for TC, especially for the seminoma subtype [26]. Recently, Paoli et al. evaluated 125 TC patients and 103 healthy controls, also finding higher consumption of milk and dairy products among cases than controls [28].

## 6. Height and Child Growth

A high calorie diet after birth could play a relevant role in TC onset [15], and adult height, which is principally determined during infancy and the first two years of life, can be assumed as a proxy of childhood diet, although stature can also be influenced by hormonal and genetic factors. Several epidemiological investigations have reported that increased adult height may be a risk factor for TC, and some propose that this relationship can be already observed in childhood, thus suggesting that environmental factors related to child growth and development may be risk factors for these cancers [18,29,30]. Other growth risk factors also related to calorie consumption, such as adult body mass index (BMI), are determined either in the infancy period or later in life, and are therefore less reliable indicators of childhood diet than height [29].

These postulations have been confirmed in recent studies. Dieckmann et al. observed that men >195 cm showed an OR of 3.35 (95% confidence intervals (CI; 2.88–3.90); adjusted) for TC compared to men under this height [29]. In the U.S. Servicemen’s Testicular Tumor Environmental and Endocrine Determinant (STEED) Study, which was a large case-control study conducted among 754 TC cases and 928 healthy controls, there was a consistent increased risk of TC associated with higher stature, particularly among men with seminomas [30]. In a hospital-based, case-control study, Giannandrea et al. reported that patients in the highest quartile of height were more likely to be diagnosed with TC than those in the lowest quartile of height (OR 2.22, 95% CIs (1.25–3.93); adjusted; *p*_trend_ = 0.033) [31]. Recently, Richiardi et al. also observed that adult height was significantly associated with TC, suggesting that environmental factors affecting growth in childhood and adolescence may therefore play an important role in that association [32]. Consequently, the trend of increasing TC incidence rates and growing stature could be due to improved diets in childhood over the past few decades.

## 7. Environmental Exposure to Endocrine-Disrupting Chemicals (EDCs) in Early Life Period

EDCs, including hexachlorobenzene (HCB), polychlorinated dibenzo-p-dioxins (PCDDs), and polychlorinated biphenyls (PCBs), are lipophilic ubiquitous compounds that were broadly used in consumer and industrial products for decades until the late 1970s [33]. These contaminants either mimic estrogens by linking estrogen receptors, or have antiandrogenic effects [34]. EDCs have long biological half-lives and their serum levels persist for decades after initial exposures in childhood. In particular, the half-life of PCBs depends on the degree of chlorination and can vary from 7–30 years [28]. As a consequence of their ubiquity and persistence, quantifiable concentrations of serum EDCs are still detected in adults in the general population today, although a recent study found that their presence has rapidly declined since 2000 [34].

Concern has recently increased about the chronic effects of these contaminants on human health, in particular for organochlorine pesticides, in particular DDT. Together with acute poisonings, there is a wide range of diseases that have been proposed to be associated with long-term exposure to organochlorine pesticides, including endocrine problems, cancer and reproductive disorders [35]. The inadequate protection of pregnant agricultural workers is an additional source of concern, due to the rising evidence that exposure to pesticides may induce reproductive impairment in a male offspring [35,36]. Owing to their durable persistence in the body, current studies on the relationship between serum concentrations of these compounds and TC have not been able to differentiate their origin, whether in the prenatal period, childhood, or later [14,37,38,39,40,41]. In addition, some recently used EDCs, such as bisphenol A (BPA) or phthalates, have been shown in animal models to be detrimental to the testes, but the available evidence in humans is still limited due to the scarce number of epidemiological studies on TC that have been so far conducted [42,43,44,45].

### 7.1. Dichloro-diphenyl-trichloroethylene (p,p’-DDE)

The agent p,p’-DDE, a metabolite of DDT, is a strong androgen receptor antagonist, which was frequently used as a pesticide, until it was banned in the 1970s [33]. Thus far, five case-control studies have measured serum levels of p,p’-DDE in relation to TC, and a significant association was observed in four of the five studies. A case-control study (49 cases, 51 controls) from the Norwegian Janus Serum Bank cohort [37], analysing individuals with confirmed TC and healthy individuals, found elevated concentrations of p,p’-DDE among cases using pre‑diagnostic serum samples. The STEED Study also detected elevated pre-diagnostic levels of p,p’-DDE among cases versus controls (OR 1.71, 95% CI (1.23–2.38), *p*_trend_ = 0.0002) [38]. Additionally, in a hospital-based study of 58 cases and 61 controls from Sweden, cases were reported to have higher blood concentrations of p,p‑DDE [39].

The one study that did not find an association between p.p-DDE levels and TC diagnosis, was the case-control study conducted by Biggs et al., where elevated concentrations of p.p‑DDE were found to not be more significantly higher among cases in respect to controls [40]. However, more recently, the fifth study, an Italian, hospital-based, case-control study, observed more elevated concentrations of p,p‑DDE in cases than controls [14], in parallel with the findings of the previous three studies.

### 7.2. Hexachlorobenzene (HCB)

The fungicide HCB has been evaluated as a risk factor for TC in three case-control studies, but none have reported evidence of a significant association [37,39,40].

### 7.3. Chlordane and Its Derivates

Three studies have measured chlordane and its derivates (oxychlordane, trans-nonachlor, cis‑nonachlor). In a Swedish, hospital-based study, higher blood levels of both trans-nonachlor (OR 4.1; 95% CI (1.5–11.0)) and cis-nonachlor (OR 3.1; 95% CI (1.2–7.8)) were detected among TC cases, in respect to healthy controls [39]. Similarly, the STEED Study also found elevated pre-diagnostic serum levels of chlordane among cases compared to controls [38]. Lastly, in the Janus Serum Bank cohort of Norway, participants diagnosed with TC cases also showed high serum concentrations of chlordane [37].

### 7.4. Polychlorinated Biphenyls (PCBs)

PCBs are a group of synthetic, persistent, lipophilic, halogenated aromatic compounds that were largely used in consumer and industrial products for several decades, before their production was forbidden in the United States in the late 1970s. PCBs were used in lubricants, cutting oils, and as electrical insulators. This group is comprised of 209 aromatic congeners, with either estrogenic or antiandrogenic activity, some of which are dioxin-like, all containing a biphenyl ring with 1–10 chlorine atoms. The IARC recently included dioxin-like PCBs in Group 1 of known human carcinogens, based on the strong evidence of the mechanism of carcinogenesis mediated by the aryl‑hydrocarbon receptor (AhR), which is identical to that of 2,3,7,8-tetrachlorodibenzo-p-dioxin (TCDD), and evidence of carcinogenicity in laboratory animals [28].

As a consequence of their wide use and persistence, PCBs still persist as ubiquitous environmental pollutants today. Measurable concentrations of blood PCBs are still found in the majority of the general population, with exposure mainly due to consumption of polluted foods (e.g., meat, fish, and dairy products) [39]. Thus far, only three studies have assessed PCB exposure in relation to TC. In a Swedish case-control study [41], no differences were found in PCB concentrations between men with or without TC. The Janus Cohort Study reported evidence that some PCB congeners, PCB congener 99 and 167 in particular, may be associated with TC risk, although the results could be influenced by the small participant numbers within the study [37]. Additionally, Paoli et al. recently observed a statistically significant increase in TC risk in cases with detectable values of total polychlorinated organic compounds versus controls (14.4% vs. 1.0%, respectively; *p* < 0.001) [28]. These initial findings suggest that the association between PCB exposure and a risk of TC is still open to further research.

## 8. Conclusions

Results of the associations between environmental childhood risk factors and TC, according to recently published epidemiological studies (2000–2015), are summarized in Table 1. TC is the most frequently occurring malignancy in adolescents and young men in the western world, and substantial effort has been expended to link differences in incidence in TC rates with reproductive, genetic, endocrine, and environmental factors. The last decades have witnessed an explosive growth in the research on TC, and current scientific research shows a possible link between environmental exposures with endocrine-disrupting activity and a risk of TC. In the early 1990s, it was suggested that increases in endogenous estrogen levels during pregnancy and/or exposure to various occupational and environmental estrogenic chemicals such as EDCs may play a causal role in the etiology of TC. Recently, it has also been suggested that exposures occurring in infancy and childhood might also increase the risk of developing TC, and that factors related to child growth and development might be deeply involved in TC progression. Although the incidence of TC has increased over the past 40 years, the reasons for this rise are still unclear. A growing number of studies have reported that increased height may be a risk factor for TC, and some suggest that this association can be already seen in childhood.TC has also been associated with childhood consumption of dairy products and with DDE serum levels.

Although suggestive, the available evidence still requires further research. Future epidemiological studies need to improve their methods of measuring human exposures in the infancy and childhood period, and new investigations should be conducted on currently used EDCs, such as plasticizers and phenols, whose reproductive toxic effects have been clearly ascertained in animal models. Finally, further research should also focus on the most appropriate life stages for examining endocrine disruptors and TC risk, in order to assess possible interactions between child development and early environmental exposures.

## Figures and Tables

**Table 1:** Summary evaluation of associations between infancy/childhood risk factors and testicular cancer (TC) according to recently published epidemiological studies (2000–2015).

Environmental Childhood Risk Factors That May Be Linked to Testicular Cancer	
Domestic and residential exposure to pesticides	+/−
Exposure to endocrine-disrupting chemicals (EDCs)	+
Height/child growth	++
Dairy consumption	+

+, significant association; +/−, inconclusive.
